# Potent Inhibitor of Human Trypsins from the Aeruginosin Family of Natural Products

**DOI:** 10.1021/acschembio.1c00611

**Published:** 2021-10-18

**Authors:** Muhammad N. Ahmed, Matti Wahlsten, Jouni Jokela, Matthias Nees, Ulf-Håkan Stenman, Danillo O. Alvarenga, Tomas Strandin, Kaarina Sivonen, Antti Poso, Perttu Permi, Mikko Metsä-Ketelä, Hannu Koistinen, David P. Fewer

**Affiliations:** † Department of Microbiology, Faculty of Agriculture and Forestry, University of Helsinki, Viikinkaari 9, Biocenter 1, P.O. Box 56, Helsinki FIN-00014, Finland; ‡ Department of Clinical Chemistry and Haematology, Faculty of Medicine, University of Helsinki and Helsinki University Hospital, Haartmaninkatu 8, P.O. Box 63, Helsinki FIN-00014, Finland; § Department of Biochemistry and Molecular Biology, Medical University in Lublin, ul. Chodzki 1, Lublin 20-093, Poland; ∥ Institute of Biomedicine and Western Cancer Centre FICAN West, University of Turku, Turku 20101, Finland; ⊥ Department of Biology, Faculty of Science, University of Copenhagen, Copenhagen DK-2100, Denmark; # Department of Virology, Faculty of Medicine, University of Helsinki, Haartmaninkatu 3, P.O. Box 21, Helsinki FIN-00014, Finland; ¶ School of Pharmacy, University of Eastern Finland, P.O. Box 1627, Kuopio FIN-70211, Finland; ∇ Dept. of Internal Medicine VIII, University Hospital Tübingen, Otfried-Müller-Strasse 14, Tübingen DE-72076, Germany; ○ Department of Biological and Environmental Science, University of Jyväskylä, P.O. Box 35, Jyväskylä FI-40014, Finland; ⧫ Department of Chemistry, Nanoscience Center, University of Jyväskylä, P.O. Box 35, Jyväskylä FI-40014, Finland; †† Department of Biochemistry, University of Turku, Turku FIN-20014, Finland

## Abstract

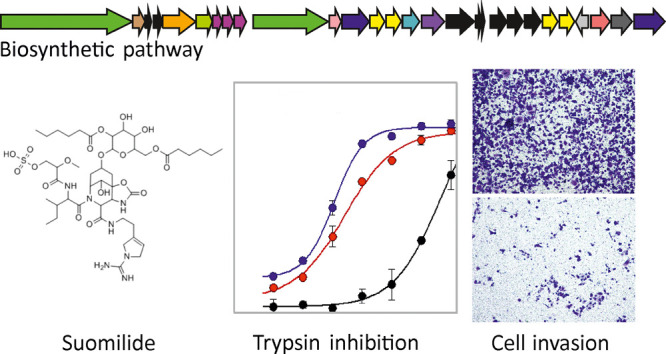

Serine proteases regulate many physiological processes and play a key role in a variety of cancers. Aeruginosins are a family of natural products produced by cyanobacteria that exhibit pronounced structural diversity and potent serine protease inhibition. Here, we sequenced the complete genome of *Nodularia sphaerocarpa* UHCC 0038 and identified the 43.7 kb suomilide biosynthetic gene cluster. Bioinformatic analysis demonstrated that suomilide belongs to the aeruginosin family of natural products. We identified 103 complete aeruginosin biosynthetic gene clusters from 12 cyanobacterial genera and showed that they encode an unexpected chemical diversity. Surprisingly, purified suomilide inhibited human trypsin-2 and -3, with IC_50_ values of 4.7 and 11.5 nM, respectively, while trypsin-1 was inhibited with an IC_50_ of 104 nM. Molecular dynamics simulations suggested that suomilide has a long residence time when bound to trypsins. This was confirmed experimentally for trypsin-1 and -3 (residence times of 1.5 and 57 min, respectively). Suomilide also inhibited the invasion of aggressive and metastatic PC-3M prostate cancer cells without affecting cell proliferation. The potent inhibition of trypsin-3, together with a long residence time and the ability to inhibit prostate cancer cell invasion, makes suomilide an attractive drug lead for targeting cancers that overexpress trypsin-3. These results substantially broaden the genetic and chemical diversity of the aeruginosin family and suggest that aeruginosins may be a source of selective inhibitors of human serine proteases.

## Introduction

Trypsins are involved in the digestion of food, but they also have important functions in many tissues outside the digestive system.^1^ The human genome encodes three trypsin isoenzymes (trypsin-1, -2, and -3) that are structurally and functionally highly similar.^1^ Trypsin-3 (also known as mesotrypsin or PRSS3) has recently been shown to promote cancer progression and metastatic dissemination in prostate, breast, pancreatic, and lung cancer.^2−5^ Thus, trypsin-3 is considered as an emerging therapeutic target for these cancers.^2−5^ Although there are subtle differences between the substrate specificities of human trypsins,^6^ the high sequence identity between trypsin isoforms extends to the active sites and makes selective targeting of trypsin-3 challenging.^7−11^ Thus, current small-molecule inhibitors of trypsin-3 target all three trypsin isoenzymes with equal potency.^11^

**Figure 1:**
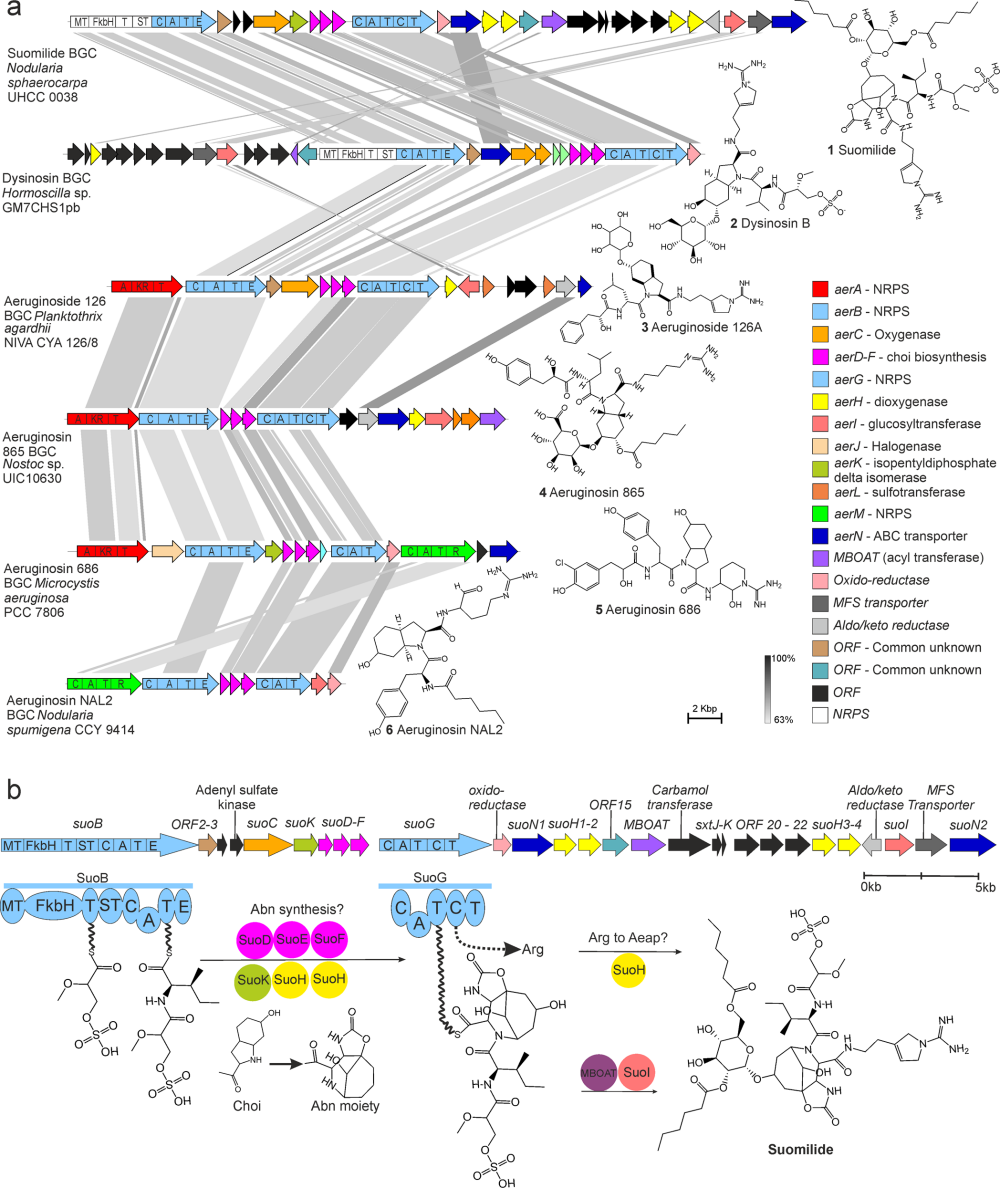
Chemical and genetic diversity in the aeruginosin family of protease inhibitors and discovery of the suomilide biosynthetic gene cluster (BGC). (a) Aeruginosin BGCs in comparison with the suomilide BGC. (b) Tentative biosynthetic scheme for the biosynthesis of suomilide.

Natural products have served as an inspiration in the development of new selective small-molecule inhibitors.^12^ Indeed, most drugs that target proteases are based on or inspired by natural products.^12^ Cyanobacteria produce an array of natural products with potent serine protease inhibition activity.^13,14^ Aeruginosins are a diverse family of linear peptides that are produced by a broad range of cyanobacteria.^14^ They are potent inhibitors of serine proteases in low micromolar to low nanomolar concentrations.^15^ Aeruginosins have a complex chemical structure characterized by the presence of an unusual 2-carboxy-6-hydroxyoctahydroindole (Choi) moiety and the C-terminal arginine derivatives argininal, argininol, agmatine, 1-amidino-2-ethoxy-3-aminopiperidine, or more rarely the 1-amino-2-(*N*-amidino-Δ^3^-pyrrolinyl)-ethyl (Aeap) moiety^16−19^ (Figure 1, panel a). The N-terminus typically consists of phenyl lactic acid derivatives^20^ but may also contain hexanoic acid^18^ or 2-O-methyl-3-sulfoglyceric acid (Mgs)^21,22^ (Figure 1, panel a). Aeruginosins may also be glycosylated, sulfonated, and halogenated.^18,19,23^ This array of structural features is responsible for their differing affinities to the catalytic binding pockets of trypsin, thrombin, and other serine proteases, as shown by the X-ray crystallography of aeruginosin–protease complexes.^15,24,25^

Aeruginosins are synthesized on nonribosomal peptide synthetase (NRPS) enzyme complexes encoded in the aeruginosin (*aer*) biosynthetic gene cluster (BGC).^18,20,22,23^ Aeruginosin biosynthesis begins with the activation of a monocarboxylic acid or fatty acid by the loading module AerA.^20,23^ AerB is responsible for adding a hydrophobic d-amino acid.^20,23^ AerD, AerE, and AerF work in a cascading manner to synthesize and supply Choi to AerG.^23,26−28^ AerG adds a Choi moiety and encodes an off-loading module.^20,23^ Variation in the loading and off-loading mechanisms of aeruginosin synthetases contributes to the observed structural diversity of the natural product family.^18−20,22,23^ Aeruginosin BGCs also encode an assortment of tailoring enzymes that promote structural diversification, including halogenation, acylation, and glycosylation.^18,19,22,23^

Suomilide is a complex tetrapeptide that contains unusual Mgs and Aeap moieties common in aeruginosins.^29^ Although suomilide lacks Choi and instead contains a densely functionalized tricyclic azabicyclononane (Abn) moiety,^29^ these structural features suggest that suomilide is a part of the aeruginosin family.^30−32^ The recent inclusion of dysinosins into the aeruginosin family further suggests that suomilide is a derived member of the aeruginosin family.^22^ However, the relationship between the suomilide and aeruginosin biosynthetic pathways has remained unclear. Suomilide, similar to many aeruginosins, has been reported to inhibit thrombin, plasmin, and trypsin at low micromolar concentrations.^29^

Here, we identify the suomilide BGC and demonstrate that suomilide belongs to the aeruginosin family of protease inhibitors. Surprisingly, we show that suomilide potently inhibits human trypsin-2 and -3, while human trypsin-1 is inhibited to a lesser degree. Importantly, our results indicate that suomilide inhibits the invasion of prostate cancer cells, making it an attractive lead molecule for the development of a drug targeting cancers associated with high trypsin-3 activity. This information should facilitate the identification of aeruginosin variants, which may facilitate future drug development studies aiming to develop even more selective trypsin-3 inhibitors.

## Results and Discussion

### Suomilide BGC

Suomilide and aeruginosins have been proposed to share a common biosynthetic logic based on the similarities in the chemical structure (Figure 1, panel a).^15,29,32^ Whole-genome sequencing of *Nodularia sphaerocarpa* UHCC 0038 yielded a complete 5.45 Mb genome organized in a single chromosome (Supporting Information Figure 1). Bioinformatics analysis identified 16 secondary metabolite BGCs (Supporting Information Figure 1 and Supporting Information Table 1), one of which showed a strong similarity to aeruginosin BGCs. The putative 43.7 kb suomilide (*suo*) BGC consisted of 28 predicted proteins, of which 14 shared similarity to gene products residing in aeruginosin BGCs (Figure 1 panel a and Supporting Information Table 2).

We propose the following biosynthetic scheme for suomilide based on earlier studies on the biosynthesis of aeruginosins.^20,23^ SuoB and SuoG encode two bimodular NRPS enzymes, which would form the suomilide backbone (Figure 1, panel b). The FkbH domain in the loading module of SuoB is likely responsible for the loading of glycerate,^33^ which would then be methylated and sulfonated (Figure 1, panel b). An identical loading module from the dysinosin biosynthetic pathway is also proposed to synthesize Mgs through the activation of glyceric acid.^22^ The second module of SuoB is predicted to activate and racemize L-Ile (Supporting Information Table 2). The first module of SuoG would activate a Choi-like compound (Figure 1 panel b and Supporting Information Table 2). The suomilide BGC encodes SuoD, SuoE, and SuoF, which are homologues of AerD, AerE, and AerF, respectively, involved in the biosynthesis of Choi.^20,23^ The suomilide BGC also encodes oxygenases (suoH1-4) and carbamoyltransferase (ORF17), which likely participate in the biosynthesis of the unique Abn moiety (Figure 1 panel b and Supporting Information Table 2). The decoration of the Abn moiety with sugar is achieved by SuoI, and the addition of hexanoic acids is likely catalyzed by membrane-bound O-acyltransferase (Figure 1 panel b and Supporting Information Table 2). The terminal off-loading module of SuoG is responsible for the addition of arginine, which is converted to Aeap through a presently unknown biosynthetic mechanism.^20^

### Genetic Diversity of the Aeruginosin Family

**Figure 2:**
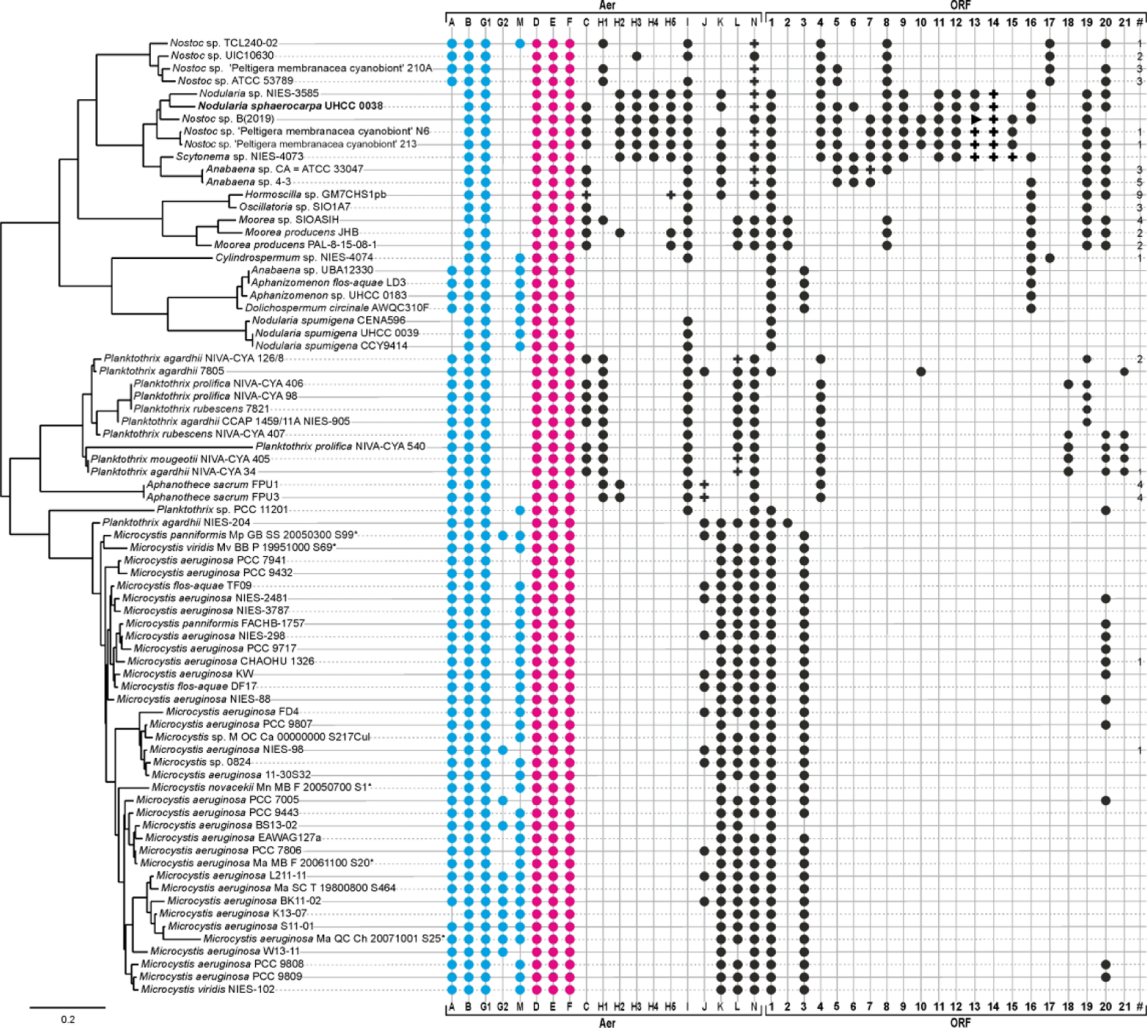
Comprehensive representation of biosynthetic gene clusters (BGCs) from the aeruginosin family of protease inhibitors. The PhyML phylogenetic tree is based on AerB, AerG, AerD, AerE, and AerF proteins from aeruginosin BGC with the scale bar representing amino acid substitutions/site. Blue dots indicate NRPS, purple dots indicate Choi-making enzymes, black dots indicate a single copy of tailoring enzymes, plus sign represents two copies of the enzymes, and a triangle represents three copies of the enzymes. The last column represents the enzymes of unknown functions and are not homologous to each other.

We examined all publicly available cyanobacterial genomes to gain insights into the genetic diversity of the aeruginosin biosynthetic pathways at the phylum level in cyanobacteria (Figure 2 and Supporting Information data set 1). We identified 103 BGCs from 12 morphologically and genetically diverse genera of cyanobacteria (Figure 2 and Supporting Information data set 1). The aeruginosin BGCs range in size from 14 to 60 kb and encode 10–31 proteins (Figure 2). We observed that AerB, AerG, AerD, AerE, and AerF proteins, which are responsible for the assembly of the peptide intermediate and synthesis of the Choi precursor, were common to all aeruginosin BGCs (Figure 2). We constructed a phylogenetic tree from concatenated sequences of five core proteins encoded in 78 BGCs (Figure 2). The suomilide biosynthetic enzymes did not form a separate clade, thus confirming the earlier predictions that this compound is part of the aeruginosin family of natural products.^30−32^

Collectively, the aeruginosin biosynthetic pathways encode three different loading mechanisms and two off-loading mechanisms (Figures 1 and 2). These allow the incorporation of diverse starter units, including phenyl lactic acid, Mgs, or hexanoic acid.^18,20,22,23^ The off-loading modules allow the release of the tetrapeptides with C-terminal arginine derivatives that include argininal, argininol, agmatine, 1-amidino-2-ethoxy-3-aminopiperidine, or more rarely an Aeap moiety.^16−19^ In addition, the aeruginosin BGCs encode a plethora of tailoring enzymes for glycosylation, sulfonation, oxygenation, halogenation, reduction, and carbamoylation of the final products (Figure 2 and Supporting Information data set 1). Our bioinformatic analyses identified many novel putative enzymes and gene combinations, suggesting that unique aeruginosin derivatives have yet to be discovered (Figure 2 and Supporting Information data set 1). This phylogenetic and genomic examination of the aeruginosin BGCs broadens the definition of the aeruginosin family of protease inhibitors to include compounds such as suomilide, dysinosin, and banyasides and suggests that the chemical diversity of the aeruginosin family is larger than currently anticipated. The selectivity of aeruginosins for different serine proteases could drive the pronounced chemical and genetic diversification of this variable family of protease inhibitors.^15^

### Suomilide Inhibits Human Trypsin Isoenzymes

**Figure 3:**
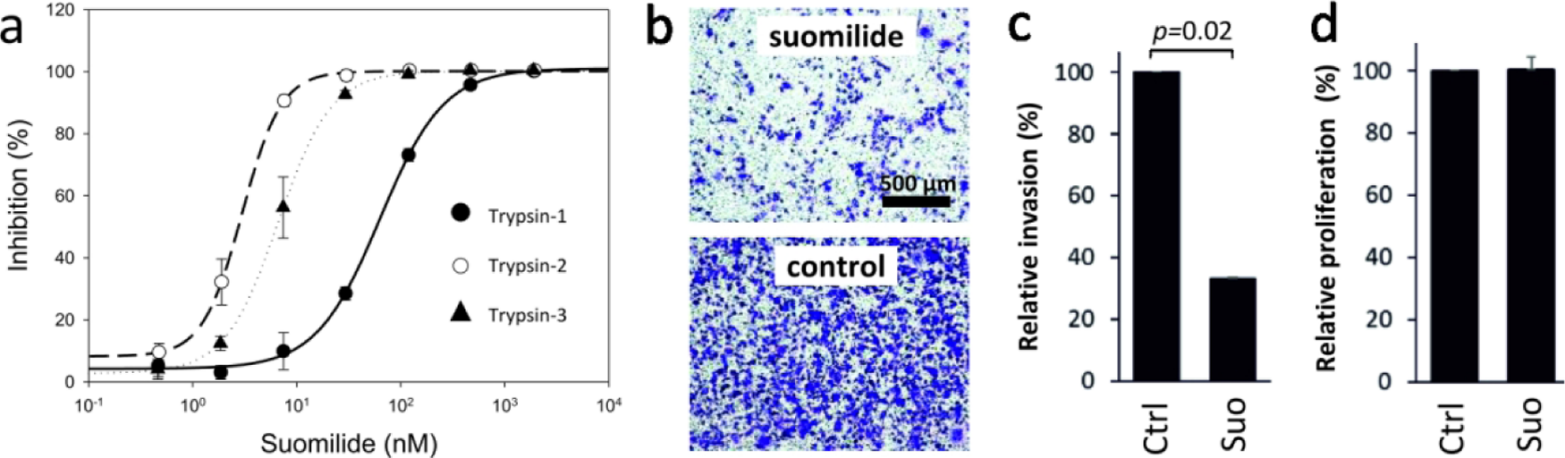
Suomilide inhibits human trypsin isoforms and PC-3M prostate cancer cell invasion. (a) Suomilide inhibits trypsin-1, -2, and -3 at IC_50_ values of 104 ± .1 ± 6.7, 4.7 ± 0.6, and 11.5 ± 2.4 nM, respectively. (b) Effect of suomilide (3.3 μM) on PC-3M prostate cancer cell invasion. (c) Representative example of staining of invaded cells (blue) (scale: 500 μm) through Matrigel basement membrane preparation in the Transwell invasion model. (d) Cell proliferation. Buffer controls (Ctrl) contained the same amount of DMSO as suomilide preparations. Average + SE of two separate experiments with four–six replicates (*n* = 2).

Suomilide has been reported to inhibit an unspecified but most likely non-human trypsin isoenzyme with an IC_50_ value of 1.8 μM.^29^ Compounds derived from cyanobacteria have most often been tested using bovine and porcine trypsins, whose primary structures differ from that of human trypsins more than human trypsins differ from each other.^34^ We purified 6.55 mg of suomilide from *N. sphaerocarpa* UHCC 0038 (Supporting Information Figures 2 and 3) and tested its ability to inhibit recombinant human trypsin-1, -2, and -3 using a chromogenic trypsin substrate. Suomilide inhibited trypsin-1, -2, and -3 with IC_50_ values of 104 ± 7, 4.7 ± 0.6, and 11.5 ± 2.4 nM, respectively (Figure 3, panel a). The preferential inhibition of trypsin-2 and -3 is notable, given the high sequence similarity between human trypsin isoenzymes that extends to the active sites, making selective targeting of individual trypsins with small molecules challenging.^11^ Indeed, suomilide shows better selectivity and potency for human trypsin-3 than the previously described small-molecule human trypsin inhibitors, such as diminazene, which equally inhibits all three human trypsins and only at micromolar concentrations.^11^ With a phage-display approach, we previously identified peptides of about 10 amino acids that show some selectivity among human trypsins, preferentially targeting trypsin-1 and trypsin-3. However, these peptides have lower potency than suomilide and also potently inhibit plasma kallikrein.^10^ As trypsin-3 has some unique features, such as Arg193 instead of Gly193 (as in bovine trypsin), it can proteolytically cleave and inactivate most natural proteinaceous trypsin inhibitors.^8^ When Gly193 in human trypsin-1 was mutated to Arg193, it conferred a trypsin-3-like resistance to inhibitors.^9,35^ On the other hand, proteinaceous inhibitors have been modified to effectively target trypsin-3.^36^ Although all human trypsins have highly similar substrate specificities, the specificity of trypsin-3 is somewhat different from those of trypsin-1 and -2, which has also allowed the creation of substrates that are preferentially cleaved either by trypsin-3 or trypsin-1 and -2.^6^ This may form a basis for the identification of more selective trypsin-3 inhibitors.

Many trypsin inhibitors also target several other trypsin-like proteases. Accordingly, we tested whether suomilide also inhibits human plasmin, plasma kallikrein, and Factor Xa. These were inhibited at IC_50_ values of 7.6 ± 2.3, 82 ± 24, and 114 ± 2 nM, respectively. However, human urokinase was inhibited at an IC_50_ value of 7.1 ± 1.36 μM, and human thrombin was minimally inhibited (IC_50_ >12.5 μM). This contrasts somewhat with the previous study,^29^ showing that suomilide inhibits undefined thrombin and plasmin with similar micromolar concentrations. Although high selectivity would be preferred for drug leads, the inhibition of plasmin may be a favorable property as plasmin is also involved in tumor progression.^37^

### Suomilide and Trypsin-3 Interaction

**Figure 4:**
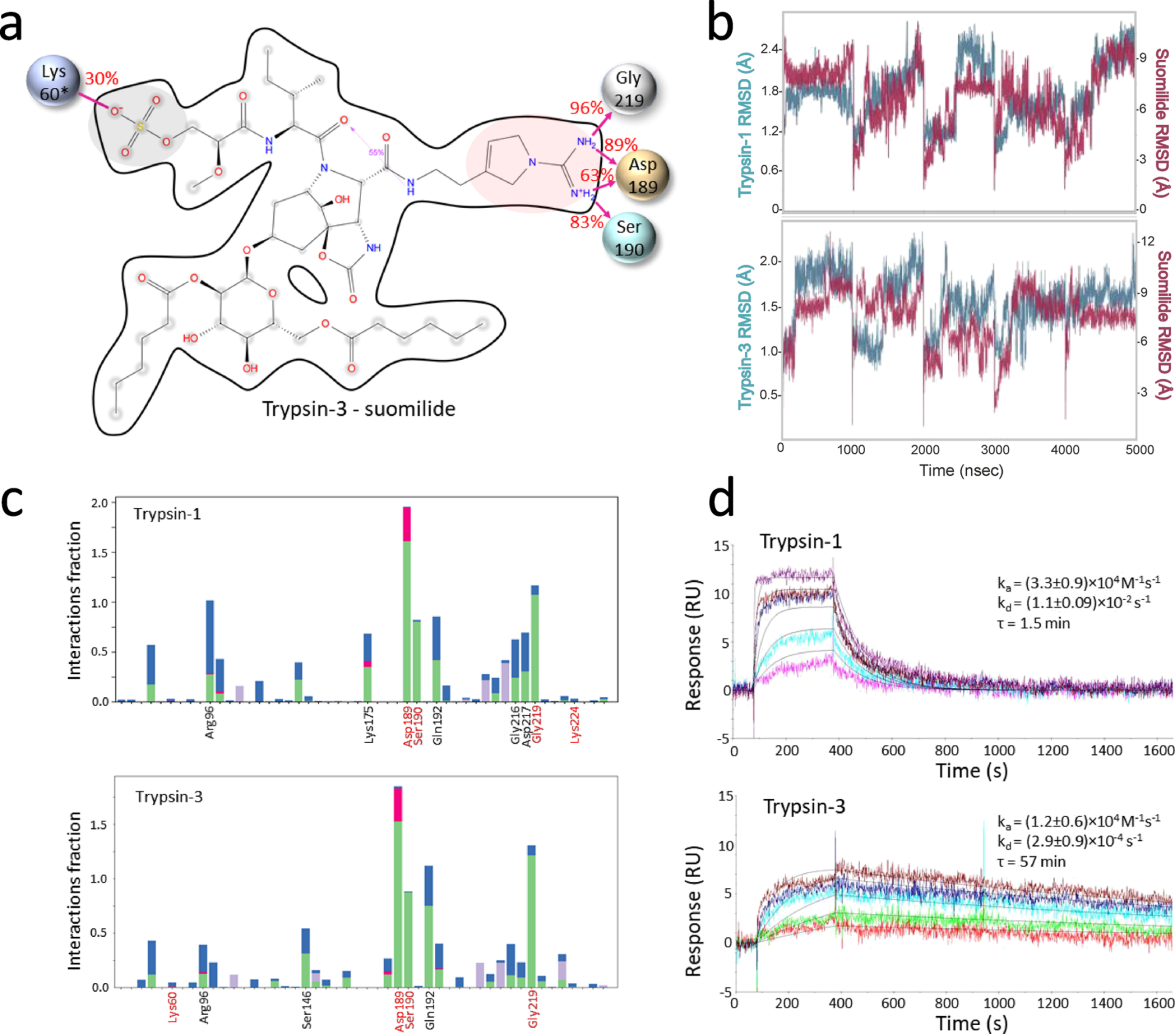
Molecular dynamics (MD) simulation of trypsin-1 and -3 interactions with suomilide and residence time. (a) Schematic of detailed ligand atom interactions with the protein residues of trypsin-3. Interactions that occur more than 30% of the simulation time (0–5004 ns) in the selected trajectory are shown. The Aeap moiety of suomilide is highlighted in red and the Mgs moiety in gray. An almost identical interaction was obtained with trypsin-1. *, Lys224 in trypsin-1. (b) Root mean square deviation plot of suomilide interaction with trypsin-1 and trypsin-3 during MD simulations. (c) Histogram of amino acids of trypsin-1 and -3 that are in contact with suomilide in MD simulation. Only amino acids whose interactions exceeded 0.5 or which are shown in (a) are shown. (d) Kinetic analysis of the suomilide interaction with trypsin-1 and trypsin-3 by surface plasmon resonance (Biacore).

To explain the selectivity of suomilide in inhibiting trypsin-2 and -3 over trypsin-1, we studied the potential binding mode of suomilide to trypsin-1 and trypsin-3, for which crystal structures are available.^7,9^ The suomilide structure was modeled, and molecular docking (Glide XP) was performed to obtain the initial docking poses. While suomilide easily docked to the trypsin-3 active site (Figure 4), trypsin-1 docking was only successful manually by using the identical binding mode as in trypsin-3. The docked complexes were then simulated for 5 × 1 microseconds. Surprisingly, for both trypsin-1 and -3, docking poses with suomilide remained almost intact throughout the simulation. In both trypsins, the hydrogen bonds between Asp189, Ser190, and Gly219 and the Aeap moiety of suomilide in particular stayed intact during the whole molecular dynamics (MD) simulation, indicating that they are important for the interaction (Figure 4, panel a and b). Asp189, which is located at the base of the specificity pocket of trypsins, has previously been found to be important also for the interaction between trypsin-3 and the trypsin inhibitor diminazene.^11^ The Mgs moiety also appeared to be important for the interaction with Lys60 in trypsin-3 or Lys224 in trypsin-1 (Figure 4, panel c). However, this interaction was not as stable as that with Aeap. These results indicate a very stable trypsin–suomilide complex (i.e., long residence time).

To experimentally validate the MD simulations, we used surface plasmon resonance, in which trypsins were immobilized on chips and binding of suomilide was followed over time. We observed that suomilide had a residence time in the range of minutes (1.5 min for trypsin-1 and 57 min for trypsin-3) with both trypsins (Figure 4, panel d). The longer residence time with trypsin-3 may explain why it was inhibited more effectively. Long residence time suggests a long-lasting drug effect, which is a desired property for many drugs, including those inhibiting the invasion of cancer cells.^38^ While molecular docking and molecular docking simulation did not provide a clear explanation for why suomilide inhibits trypsin-2 and -3 more potently than trypsin-1, these analyses were partially able to explain the strong inhibitory potency of suomilide.

### Suomilide Inhibits the Invasion of Prostate Cancer Cells

Trypsin-3 has gained considerable interest as a potential drug target. Trypsin-3 is overexpressed in various cancers and appears to drive cancer progression.^2−5^ We tested the bioactivity of suomilide using a Transwell cell invasion assay, in which the invasion of cells through a Matrigel basement membrane preparation was studied. In this model, 3.3 μM suomilide significantly inhibited the invasion of trypsin-3-producing aggressive metastatic PC-3M prostate cancer cells.^39^ Suomilide did not affect the proliferation of cells grown on a plastic surface (Figure 3, panels b-d). The model was chosen based on previous studies, indicating that that trypsin-3 is an important mediator of prostate cancer progression and metastasis.^3,40^ In these previous studies, the role of trypsin-3 in prostate cancer was shown using a mouse orthotropic model and different cell models. These included PC-3M cells, whose invasion was reduced upon the knockdown of trypsin-3 expression by RNA interference and a modified proteinaceous trypsin-3 inhibitor.^3,40^ Of note, the knockdown of trypsin-1 in PC-3M cells did not have such an effect.^3^ Thus, cell invasion in this model appears to be regulated by the trypsin-3 activity. The functional studies support the potential of suomilide as a lead compound for the development of an anti-invasive cancer drug. Targeting invasion is important as it is a prerequisite of the metastatic dissemination of cancer cells, and most cancer-associated deaths in patients with solid cancers (such as prostate cancer) are caused by metastases.^41^

## Conclusions

We explored the biosynthetic diversity encoded in aeruginosin biosynthetic pathways using whole-genome sequencing and bioinformatics analyses and confirmed the long-standing hypothesis that aeruginosins and suomilide are members of the same family of natural products. The overall genetic diversity of the aeruginosin biosynthetic gene clusters suggests that the chemical and genetic diversity is greater than currently appreciated. We observed that suomilide is a potent inhibitor of human trypsin-2 and -3 isoenzymes and also inhibits the invasion of PC-3M prostate cancer cells without affecting proliferation. Therefore, in addition to showing that suomilide is a potential lead for the development of anti-invasive drugs, in a broader context, this suggests that aeruginosins are a good source of lead compounds for the further development of protease inhibitors.

## Methods

### Genome Sequencing

The cyanobacterial strain *N. sphaerocarpa* UHCC 0038 (formerly HKVV) was grown in a photon irradiance of 15 μmol m^–2^ s^–1^ in 100 mL of Z8XS culture medium, obtained by adding 0.88% NaCl and 0.38% MgSO_4_·7H_2_O to Z8 media,^42^ for 25–30 days at 21–25 °C. Genomic DNA was isolated using an E.Z.N.A. SP Plant DNA extraction and purification kit (Omega Bio-Tek). The culture was centrifuged at 7000*g* for 7 min (Sorvall LYNX 6000 superspeed centrifuge, Thermo Fisher Scientific). The supernatant was discarded, and the pellet was transferred to a 2 mL tube. 100 μL of 425–600 μm beads and 100 μL of 700–1180 μm of beads (Scientific Industries) were added to the pellet after addition of 600 μL of SP1 buffer (provided with the kit) to 50 mg of cells. The cells were mechanically broken using a Fastprep cell disrupter (Bio 101, Thermo Electron Corporation, Qbiogene, Inc.) at a speed of 6.5 ms^–1^ for 20 s, repeating this step twice. 5 μL of RNAse buffer (provided with the kit) was added prior to vortexing. The remainder of the procedure was followed as indicated in the kit manual. The purification yielded 1616 μg of genomic DNA. The quality and concentration of isolated DNA samples were assessed using an Agilent TapeStation (Agilent Technologies) and a NanoDrop 1000 spectrophotometer (Thermo Scientific), respectively.

Whole-genome sequencing was performed using a combination of PacBio RSII and Illumina MiSeq, both reaching an average of 77× coverage. Nextera adapters were removed from Illumina data by cutadapt v1.14, keeping reads with a minimum of 50 bp length and Phred 25 quality. PacBio data were assembled with HGAP3, implemented in SMRTportal 2.3. Illumina data were assembled with SPAdes v3.11.1 with the “careful” option. Gap4 was used to combine assemblies and check sequence circularity. Illumina reads were mapped against the combined assembly with bwa 0.7.12-r1039. The genome was then polished using Pilon v1.16 to fix homopolymer errors. Finally, the complete sequence was annotated using Prokka v1.13, and protein functional annotation was performed with PANNZER2. All sequencing, assembly, and annotation were performed at the DNA Sequencing and Genomics laboratory, Institute of Biotechnology, University of Helsinki. The complete genome sequence of *N. sphaerocarpa* UHCC 0038 was deposited in the GenBank database under the accession numberCP060140.

### Identification of Aeruginosin BGCs

AerD, AerE, AerB, and AerG from the aeruginosin BGC of*Planktothrix agardhii*NIVA-CYA 126/11 were used to query the non-redundant (*nr*) database at NCBI in BLASTp searches. The identified BGCs were manually annotated using Artemis version 18.1. BLASTp searches were performed on the GenBank database (release no. 242.0) to predict the function of genes present in the BGCs; the BGCs were then annotated manually. The conserved domains (CD) in the NRPS and the function of the genes were also predicted by using the NCBI CD Database (CDD). The substrate specificity of the NRPS adenylating domains was predicted using the NRPSpredictor2 online version and CDD searches. antiSMASH 6.0 analysis was performed to identify the BGCs in the genome of *N. sphaerocarpa* UHCC 0038. Easyfig (version 2.2.2.) was used to compare and draw the BGCs of *N. sphaerocarpa* UHCC 0038, *Hormoscilla* sp. GM7CHS1pb,*P. agardhii*NIVA-CYA 126/8,*Nostoc* sp. UIC 10630,*Microcystis aeruginosa* PCC 7806, and*Nodularia spumigena*CCY9414.

### Phylogenetic Trees

Phylogenetic trees based on suomilide and aeruginosin biosynthetic proteins were constructed using PhyML to test the relationship between suomilide and aeruginosin.^43,44^ An alignment was constructed combining the AerB (CATE domains only), AerG (CAT domains only), AerD, AerE, and AerF proteins obtained from the suomilide and aeruginosin BGCs. The ClustalW multiple sequence alignment was constructed using BioEdit (version: 7.0.5.3). The phylogenetic trees, constructed using the “advanced” model, relied on sequence homology.^43^ Using this model, we skipped the multiple sequence alignment as this was performed using BioEdit. The curation of the aligned sequences was performed with the Gblocks program, which excluded the ambiguous regions in the aligned sequences. PhyML program was used for the construction of the trees. Visualization of the phylogenetic trees was performed by TreeDyn software.

### Chemical Analysis

The biomass of *N. sphaerocarpa* UHCC 0038 was harvested from 40 mL cultures through centrifugation at 7000*g* for 7 min (Sorvall LYNX 6000 superspeed centrifuge, Thermo Fisher Scientific) and was subjected to freeze-drying (Christ Beta 2–8 LSCplus, LyoCube 4–8) for 72 h, yielding 10–50 mg of the dried sample. The freeze-dried sample was extracted with 1 mL of 70% methanol [HiperSolv for high-performance liquid chromatography (HPLC), BDH Laboratory supplies], 200 μL of 0.5 mm glass beads (Scientific Industries) was added, and cells were mechanically broken using a FastPrep cell disrupter (Bio 101, Thermo Electron Corporation, Qbiogene, Inc.) at a speed of 6.5 ms^–1^ for 30 s. The suspension was centrifuged at 20 000 rpm for 5 min (Eppendorf Centrifuge 5804R, Eppendorf AG) at room temperature and was stored at 4 °C for LC–mass spectrometry (LC–MS) and quadrupole time-of-flight (QTOF) analyses.

### Liquid Chromatography–Mass Spectrometry

100 μL of the MeOH extract was filtered through a 0.2 μm filter (13 mm syringe filter, PTFE, VWR International). Sample analysis was performed with an ultra-performance liquid chromatograph interfaced to a high-definition electrospray ionization mass spectrometer [Acquity ultra-performance LC (UPLC) I-Class-Synapt G2-Si Q-TOF, Waters]. A Kinetex C8 (50 × 2.1 mm, 1.7 μm 100 Å, Phenomenex) column was used for separation. The mobile phase consisted of a 0.1% aqueous solution of formic acid (A) and 0.1% formic acid in a 1:1 mixture of acetonitrile and 2-propanol (B). The following gradient was run: from 5 to 100% B in 5 min, maintained at 100% B for 2 min, and then back to 5% B in 30 s where it was maintained for 2.5 min. The total run time was 10 min. The injection volume was 1 μL. The instrument parameters for MS and MS/MS analysis were a capillary voltage of 1.5 kV, a source offset of 80.0, a source temperature of 120 °C, a sampling cone of 40.0, a desolvation temperature of 600 °C, a desolvation gas flow of 1000 L h^–1^, an ion energy of 1.0, a nebulizer gas pressure of 6.5 bar, a trap wave velocity of 300.0 V, and a trap collision energy of 30.0.

### Purification of Suomilide

*N. sphaerocarpa* UHCC 0038 was grown in 210 L of Z8XS culture medium, centrifuged at 7 000*g* for 7 min and then lyophilized (Christ Beta 2–8 LSCplus, LyoCube 4–8) for 72 h, yielding 30 g of dried biomass. The dried cells were lyophilized, and 5 g was lysed in 60 mL of methanol using a SilentCrusher M homogenizer (Heidolph) for 30 s at 15 000 rpm. After the centrifugation at 10 000*g* for 5 min, the supernatant was collected, and the extraction was repeated. The crude extract (120 mL) was rotary-evaporated (Büchi) to dryness in 5 g of octadecylsilyl silica gel (Fuji-Davison Chemical Ltd.) at 150 mbar at 25 °C. The silica gel-bound sample was fractionated in two batches using a solid-phase Strata SI-1 silica cartridge 5 g/20 mL (Phenomenex). The sample was loaded on a preconditioned cartridge and sequentially eluted with *n*-heptane, acetonitrile, acetone, ethyl acetate, and methanol. The fractions were analyzed with the aforementioned UPLC–QTOF system. The methanol fractions that contained suomilide were dried under a nitrogen gas stream, and suomilide was purified by semi-preparative HPLC combined with MS using a Luna C8(2) (150 × 10.00 mm, 5 μm, Phenomenex) column and a Waters Auto Purification System. The positive-ion mode was used in the electrospray ionization. The sample was dissolved in 2 mL of the eluent, and the injection volume was 150 μL with a flow rate of 5 mL min^–1^. The mobile phase consisted of 0.1% aqueous formic acid (solvent A) and acetonitrile/isopropanol (1:1) (solvent B). A gradient from 30 to 100% of solvent B over 7 min was used, and the total run time was 14 min. The organic solvents were evaporated from the fractions under a nitrogen gas stream. The fractions were dissolved in methanol and pooled together to be analyzed with the aforementioned UPLC–QTOF system. Analysis showed that the suomilide preparation contained only very minor amounts of other suomilide variants (Supporting Information Figure 2). The molecular formula of C_45_H_73_N_7_O_19_S was established based on UPLC–QTOF analysis (Supporting Information Figure 2). *N. sphaerocarpa* UHCC 0038 does not synthetize secondary metabolites such as nodularins, spumigins, aeruginosins, and nodulapeptins,^18,44^ typical for*N. spumigena* strains and some other*Nodularia* species (Supporting Information Figure 3). To further assess the purity, suomilide was evaporated to dryness and subjected to nuclear magnetic resonance (NMR).

### Concentration and Purity Determination through NMR

The purification yielded 7.7 mg of suomilide from *N. sphaerocarpa* UHCC 0038, as determined by weighing. Suomilide was dissolved in 0.5 mL of DMSO-*d*_6_ (dimethyl sulfoxide). Commercial dipeptide Tyr–Val (Sigma-Aldrich) was dissolved to 1/10 of the volume of the suomilide solution in an equimolar ratio with suomilide. The comparison of integrated proton spectrum signals from suomilide and Tyr–Val dipeptide gave a 10% lower concentration for suomilide than that obtained by weighing. ^13^C HSQC (heteronuclear single quantum coherence) showed several minor signals in the carbohydrate region in addition to two minor signals in the anomeric region (Supporting Information Figure 4). This indicates that the suomilide preparation contains suomilide variants in which fatty acid residues are different or located in different positions in the glucose unit. The comparison of suomilide and variant signal intensities yielded an estimate of 85% suomilide and 15% suomilide variants. The weight determined by NMR (6.55 mg) was used in this study.

One-dimensional ^1^H and two-dimensional CH_*n*_-edited ^13^C-HSQC NMR spectra were measured in a Bruker-AVANCE III HD 800 NMR spectrometer, equipped with a cryogenically cooled triple-resonance (^1^H, ^13^C, and ^15^N) probe head and *z*-gradient at 30 °C (303 K). For ^1^H spectra, 16 k complex points, corresponding to an acquisition time of 1.28 s, were collected using 16 transients. ^13^C-HSQC spectra were collected with 24 transients per FID and 256/1024 complex points in *t*_1_/*t*_2_, corresponding to acquisition times of 7.1 and 92 ms, respectively.

### Serine Protease Inhibition

Recombinant trypsinogens-1, -2, and -3 were produced in*Escherichia coli*and activated to trypsin-1, -2, and -3 as described previously.^45^ The enzyme inhibitory activity of suomilide was determined in 96-well plates by adding 10 μL of diluted suomilide (0.21–3400 nM, diluted in ultra-purified water, containing up to 0.027% DMSO), 15 μL of 0.1% bovine serum albumin in 50 mM Tris-buffer saline (BSA/TBS), and 25 μL of each trypsin (1.88 nM). 10 μL of ultra-purified water was used as a control. DMSO at the concentrations used did not have any effect on the activity of trypsins (data not shown). Preincubation of suomilide with trypsin isoenzymes prior to the addition of the substrate was required for maximal inhibition (data not shown). Thus, suomilide was preincubated with trypsins at room temperature for 30 min before the addition of 50 μL of 0.2 mM chromogenic substrate S-2222 (ChromogeniX) in ultra-purified water. The change of absorbance at 405 nm was followed for 15 min (VICTOR X4 multilabel plate reader, PerkinElmer). The experiment was performed three times, each with two replicates (*n* = 3). The absorbance change during the phase of the substrate reaction in which the absorbance of the control reaction increased linearly over time was determined. The same experiment was performed with plasmin (Sigma) (15 nM) with chromogenic substrate S-2251 (ChromogeniX); plasma KLK (Sigma) (20 nM) with substrate ChromogeniX S-2302 (ChromogeniX); Factor Xa (Sigma) (3 nM) with substrate S-2222; and thrombin (Sigma) (1 nM) with substrate S-2238 (ChromogeniX). Except for urokinase from human kidney cells (Sigma) (125 IU), 15 μL of BSA/TBS with 0.9% NaCl was used. Nine 4-fold dilutions of suomilide were prepared, with 12.5 μM being the highest final concentration. The IC_50_ values were estimated using Quest Graph, and the standard error was calculated using MS Excel 2013.

### Effect of Suomilide on the Invasion and Proliferation of PC-3M Cells

PC-3M prostate cancer cells^46^ were cultured in Ham’s F12 Nutrient Mix with GlutaMAX (Gibco), supplemented with 10% fetal calf serum (FCS), 100 IU mL^–1^ penicillin, 100 μg mL^–1^ streptomycin, and 0.15% sodium bicarbonate at 37 °C in a humidified, CO_2_-controlled (5%) incubator. For the invasion assay, PC-3M cells (1.45 × 10^5^) in a serum-free medium containing 0 or 3.3 μM suomilide were added into polycarbonate membrane Transwell inserts with 8.0 μm pore size (Thermo Fisher) covered with 100 μL of the phenol red-free Matrigel basement membrane matrix (Corning) diluted 1:30 with phosphate-buffered saline. The inserts were placed into a 24-well plate containing 750 μL of the culture medium with 10% FCS and incubated for 4 days at 37 °C in a humidified, CO_2_-controlled (5%) incubator. Matrigel and the noninvasive cells were removed using cotton swabs, and the membranes were stained with 0.09% crystal violet for 10 min. The experiment was performed twice, both with four replicates (*n* = 2). The invasion was quantitated from microscopic images (three panels from each of the replicates) using Fiji ImageJ. For the proliferation assay, 7500 cells/well were cultured as above on a 96-well plate with 0 or 3.3 μM suomilide in 100 μL of culture media containing 1% FCS. After 3 days, 10 μL/well CCK-8 solution (Cell Counting Kit-8, Dojindo) was added, and absorbance at 450 nm was measured after 1.5 h incubation. The experiment was performed twice, both with six replicates (*n* = 2). *T*-Test (two-tailed, Microsoft Excel) was used to assess the differences in the invasion and proliferation between suomilide-treated and control cells.

### Molecular Modeling

All modeling was conducted with Maestro Small-Molecule Drug Discovery Suite 2021-1 (Schrödinger Release 2021-1: Maestro, Schrödinger, LLC, 2021) with an OPLS4 force field unless otherwise stated.^47^ The figures were prepared with PyMOL 2.4.2 (Schrödinger, LLC). For the docking and MD simulations, we used the PDB structures 2RA3 (trypsin-1)^7^ and 1H4W (trypsin-3),^9^ which were prepared using Protein Preparation Wizard (default settings with the “cap termini” option).^48^ The amino acid numbering used here is based on the numbering in these PDB structures. The initial coordinates for the ligand–protein complexes were obtained by Glide XP docking using default settings.^49−51^ The grid box was defined by SiteMap (the SiteMap region covering the active site was selected for GRID).^52^ Before docking, suomilide was prepared with LipPrep (default settings) using Epik, and the tautomeric state of suomilide was based on the QM Conformer & Tautomer Predictor results. The MD simulations were conducted with Desmond.^53^ The systems were solvated in a cubic box (edges 14 Å from the protein) and neutralized with counter ions (2 Cl^–^) with 0.15 M NaCl salt. The water was described with the SPC water model. The final trypsin-1 and trypsin-3 systems consisted of 33 840 and 36 545 atoms, respectively. The default relaxation protocol of Desmond was used before the 1000 ns production simulations, which were conducted in the NPT ensemble (300 K, thermostat: Nosé–Hoover chain; 1.01325 bar, barostat: Martyna–Tobias–Klein). The default time step of 2 fs and cutoff radius of 9.0 Å for Coulombic interactions were used. The total simulation time was 5 μs (5 × 1000 ns) for both systems. All the MD setup and trajectory files and films are available at Zenodo (https://zenodo.org/record/5512048#.YVF1w-dRVaQ).

### Surface Plasmon Resonance

The binding of suomilide to trypsin-1 and -3 was analyzed with surface plasmon resonance in Biacore 2000 instrumentation (Cytiva). The flow cells of a CMD700M biosensor chip (Xantec bioanalytics) were covalently coated with trypsin-1, trypsin-3, or a proteolytically inactive trypsin-3 mutant (negative control) via standard amine coupling. The coating resulted in a resonance unit increase of 5600–7200. The binding was analyzed using Tris-buffered saline (pH 7.4) containing 0.01% DMSO as the running buffer and a flow rate of 30 μL min^–1^. The kinetics of the suomilide interaction with the trypsins were measured by varying suomilide concentrations from 250 to 4000 nM with a contact time of 5 min and a dissociation phase of 20 min. After the completion of the dissociation phase, the flow cells were regenerated with 10 mM Glycine, pH 1.7. The data were evaluated by first subtracting the sensorgrams obtained from the trypsin-3 mutant control flow cell from those obtained from the flow cells containing trypsin-1 or -3 for each suomilide concentration separately and overlaying the resulting sensorgrams for each ligand. Association and dissociation rate constants were obtained by the Langmuir global fit model (BiaEvaluation Software, Cytiva). The residence time was calculated as a reciprocal of the dissociation constant.

## Supporting Information Available

103 aeruginosin BGCs identified through the bioinformatic analysis. A link for molecular dynamic simulations: https://zenodo.org/record/5512048#.YVF1w-dRVaQ (XLSX)
Analysis of polyketide synthetases and NRPS BGCs in the complete genome of *N. sphaerocarpa* UHCC 0038, UPLC–QTOF purity analysis of suomilide preparation, total ion chromatograms of positive and negative ionizations of the *N. sphaerocarpa* UHCC 0038 methanol extract, ^1^H and edited ^13^C HSQC spectra of suomilide, antiSMASH analysis of the *N. sphaerocarpa* UHCC 0038 genome, and function prediction of the suomilide biosynthetic gene cluster from *N. sphaerocarpa* UHCC 0038 and comparison between suomilide and aeruginosin biosynthetic gene cluster proteins (PDF)

## Accession Codes

The accession number of the complete genome sequence of *N. sphaerocarpa* UHCC 0038: CP060140.
