# Supplementary material for: Potent Inhibitor of Human Trypsins from the Aeruginosin Family of Natural Products
Source: ACS Chem Biol. 2021 Oct 18;16(11):2537–46. doi: 10.1021/acschembio.1c00611 (PMC8609519; doi:10.1021/acschembio.1c00611)
Supplement: Supplementary file 2 — cb1c00611_si_002.pdf [file cb1c00611_si_002.pdf]

Supplementary Information for

A potent inhibitor of human trypsins from the aeruginosin family of natural products

Muhammad N. Ahmed[1,2], Matti Wahlsten[1], Jouni Jokela[1], Matthias Nees[3,4], Ulf Håkan Stenman[2], Danillo O. Alvarenga[1,5], Tomas Strandin[6], Kaarina Sivonen[1], Antti Poso[7,8], Perttu Permi[9,10], Mikko Metsä-Ketelä[11], Hannu Koistinen[2]*, and David P. Fewer[1]*

[1]Department of Microbiology, Faculty of Agriculture and Forestry, P.O. Box 56, Viikinkaari 9, Biocenter 1, FIN-00014 University of Helsinki, Finland.
[2]Department of Clinical Chemistry and Haematology, Faculty of Medicine, P.O. Box 63 (Haartmaninkatu 8) FIN-00014 University of Helsinki and Helsinki University Hospital, Finland.
[3]Department of Biochemistry and Molecular Biology, Medical University in Lublin, ul. Chodzki 1, 20-093 Lublin, Poland.
[4]Institute of Biomedicine and Western Cancer Centre FICAN West, University of Turku, 20101 Turku, Finland.
[5]Department of Biology, Faculty of Science, University of Copenhagen, DK-2100, Copenhagen, Denmark
[6]Department of Virology, Faculty of Medicine, P.O. Box 21 (Haartmaninkatu 3) FIN-00014 University of Helsinki
[7]School of Pharmacy, University of Eastern Finland, P.O. Box 1627, FIN-70211 Kuopio, Finland
[8]University Hospital Tübingen, Dept. of Internal Medicine VIII, Otfried-Müller-Strasse 14, DE-72076 Tübingen, Germany
[9]Department of Biological and Environmental Science, University of Jyväskylä, P.O Box 35, FI-40014 Jyväskylä, Finland.
[10]Department of Chemistry, Nanoscience Center, University of Jyväskylä, P.O Box 35, FI-40014 Jyväskylä, Finland.
[11]Departments of Biochemistry, University of Turku, FIN-20014 Turku, Finland

Corresponding authors: David P Fewer, Hannu Koistinen
Email: david.fewer@helsinki.fi, hannu.k.koistinen@helsinki.fi

This file includes:

Supplementary text

Supplementary Figure 1 to 4

Supplementary Table 1 to 2

Other supplementary materials for this manuscript include the following:

Supplementary Dataset 1

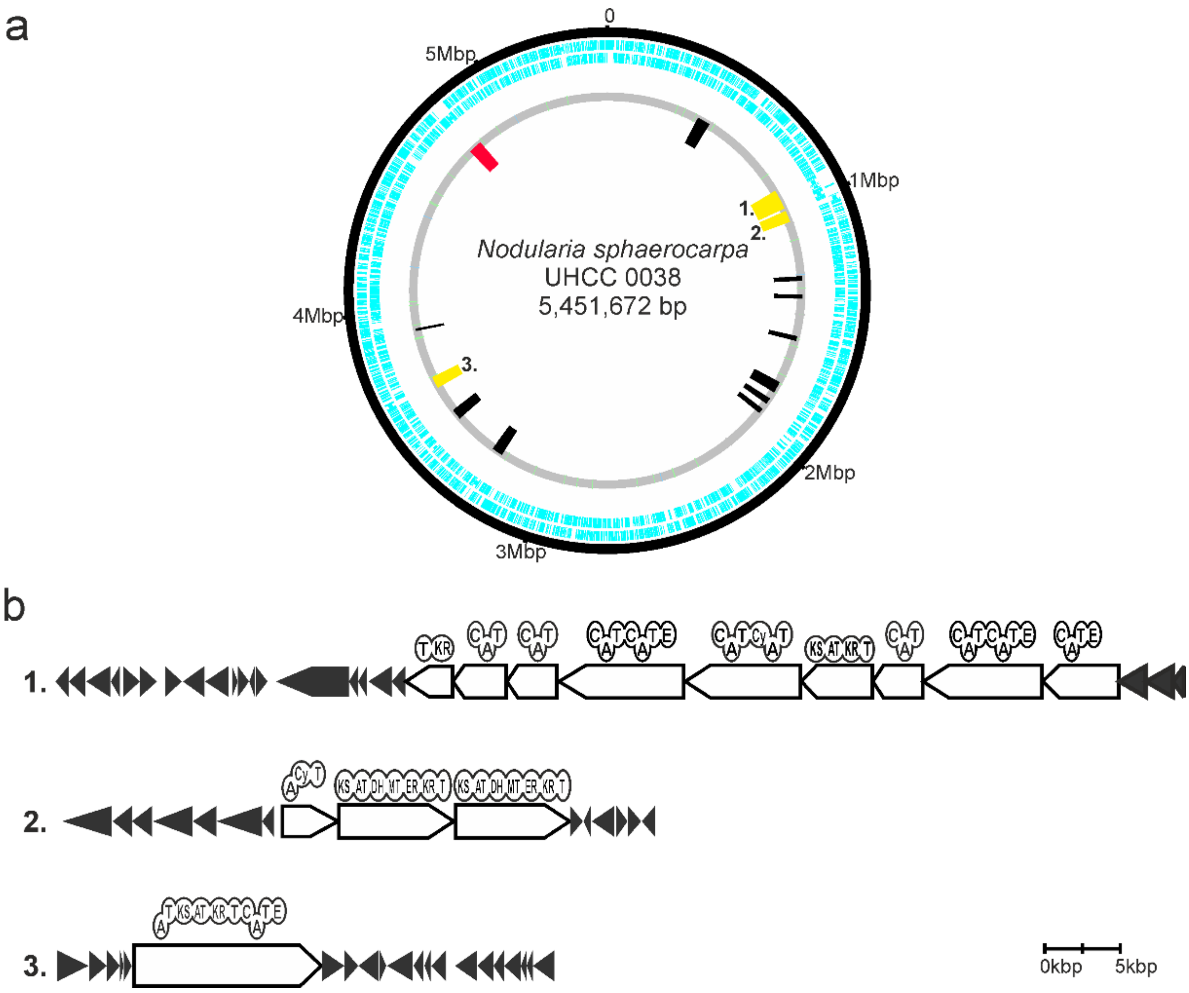

Supplementary Figure 1. Bioinformatic analysis of non-ribosomal peptide synthetase (NRPS) and polyketide synthase (PKS) biosynthetic gene clusters (BGCs) encoded in the complete genome of *Nodularia sphaerocarpa* UHCC 0038 performed using antiSMASH 6.0 (a) Circular map of the complete genome, NRPS/PKS BGCs are marked in yellow, the suomilide BGC is marked in red and other secondary metabolite BGCs are marked in black (b) The three BGCs predicted to encode mixed NRPS/PKS peptides and the annotation of their catalytic domains.

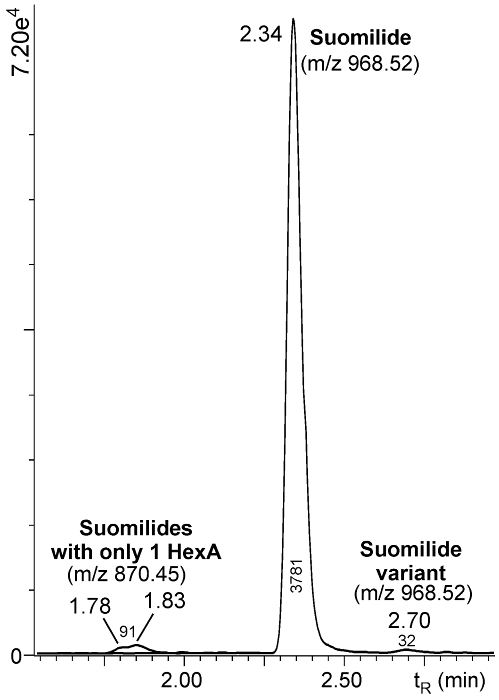

Supplementary Figure 2. Ultra-high performance liquid chromatography-quadrupole time-of-flight mass spectrometry (UPLC-QTOF) analysis of purified suomilide. Extracted ion chromatograms of suomilide (retention time 2.34 min, *m/z* 968.52) and a minor suomilide variant (retention time 2.70 min, m/z 968.52), and suomilide variants with one hexanoic acid (HexA) (retention times 1.78 and 1.83, m/z 870.45) (all suomilides without sulfate group). Peak areas (numbers inside the peaks) show that suomilide (retention time 2.34, m/z 968.52) is practically free of other suomilide variants.

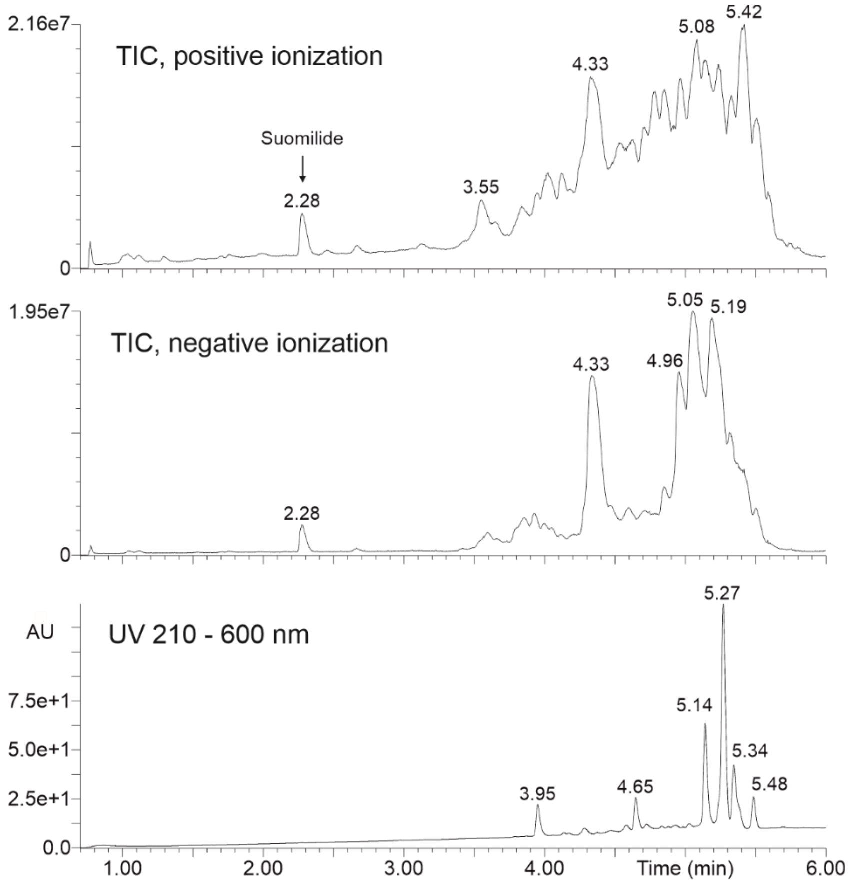

Supplementary Figure 3. Total ion chromatograms (TIC) with positive and negative ionizations of *Nodularia sphaerocarpa* UHCC 0038 methanol extract. No other major compounds eluted near suomilide. Suomilide is not visible In the UV chromatogram, because it does not contain structural elements with absorption between 210–600 nm.

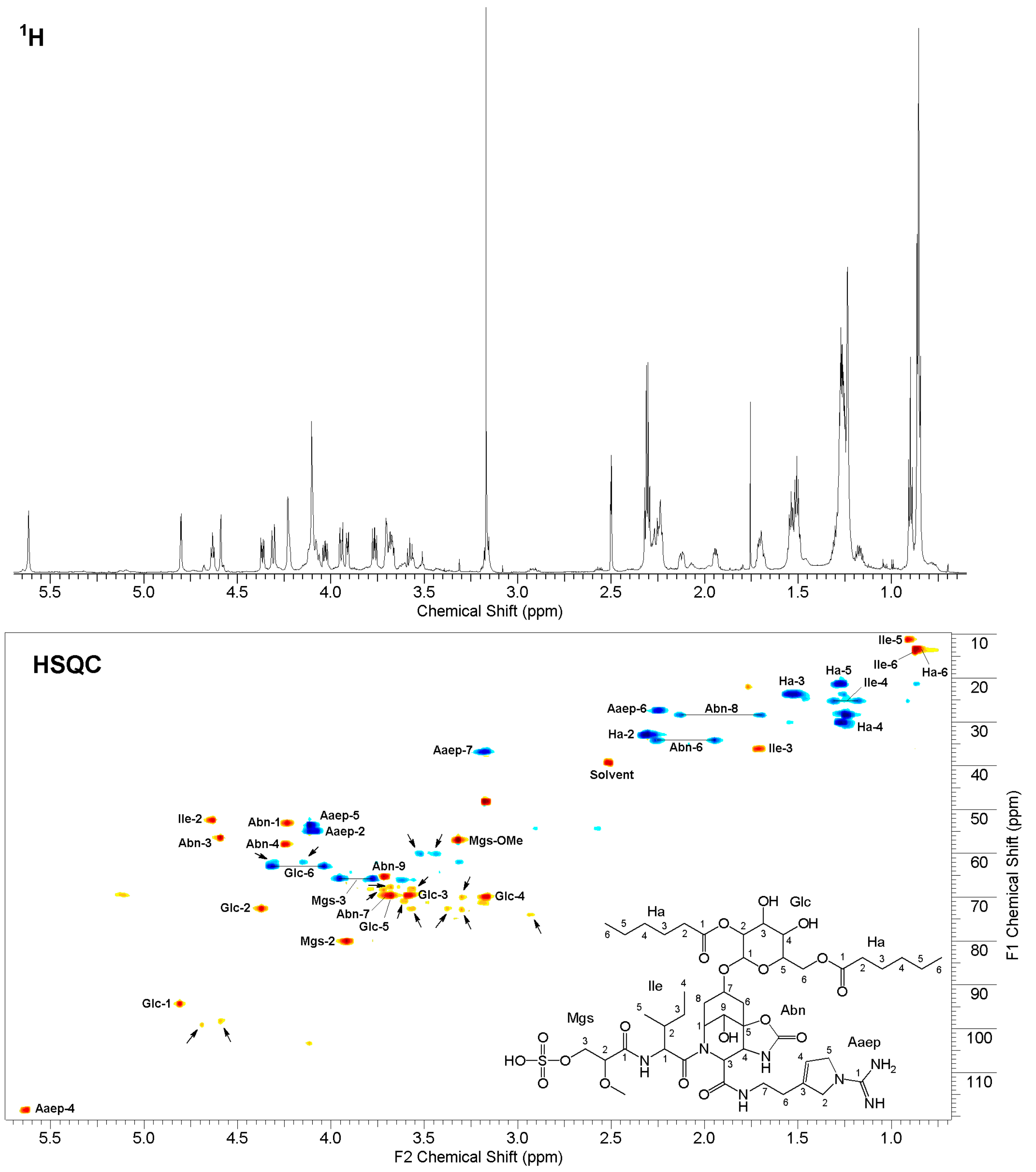

Supplementary Figure 4. ^{1}H spectrum and edited ^{13}C HSQC spectrum of suomilide. Arrows show low intensity signals at carbohydrate region representing minor suomilide variants, which probably differ in respect of fatty acid residue length/position in glucose (Glc).

Supplementary Table 1. AntiSMASH 6.0 analysis of the *Nodularia sphaerocarpa* UHCC 0038 genome. Abbreviations: PKS, Type I polyketide synthase; NRPS, Non-ribosomal peptide synthetase; hgIE-KS, heterocyst glycolipid synthase-like polyketide synthase.

BGC	Cluster type	Size (kb)	Most similar known cluster	Similarity (%)
1	Lassopeptide or bacterocin	58.1	Yersiniabactin	2
2	PKS, NRPS	88.1	Nostophycin	27
3	PKS, NRPS-like	51.3	Puwainaphycin F/minutissamide A	33
4	Microviridin	20.2	Microviridin K	37
5	Lanthipeptide-class-II	18.5	-	-
6	Lanthipeptide-class-V	39.4	-	-
7	Terpene	20.9	-	-
8	hgIE-KS, T1PKS	51.8	Heterocyst glycolipids	100
9	Polybrominated diphenyl ether	26.7	-	-
10	Lanthipeptide-class-ii	23.3	-	-
11	Lanthipeptide-class-v	42.5	Anacyclamide A10	21
12	NRPS-like, PKS	48.8	-	-
13	hglE-KS	47.2	Heterocyst glycolipids	57
14	PKS, NRPS	52.2	-	-
15	Bacteriocin	10.2	-	-
16	NRPS	61.6	Heterocyst glycolipids	47

Supplementary Table 2. Functional prediction of the suomilide biosynthetic gene cluster (BGC) proteins from *Nodularia sphaerocarpa* UHCC 0038 and comparison between suomilide BGC proteins and aeruginosin BGC proteins from *Hormoscilla* sp. GM7CHS1pb, *Planktothrix agardhii* NIVA-CYA 126/8, *Microcystis aeruginosa* PCC 7806, and *Nodularia spumigena* CCY9414 and *Nostoc* sp. UIC 10630.

Protein	Amino acids	Predicted function	Pairwise sequence identity compared to aeruginosin BGCs (%)					
			Aer	GM7CHS1pb	126/8	PCC7806	CCY9414	UIC10630
SuoB	2875	Non-ribosomal peptide synthetase	AerB	-	56	589	46.2	62.6
ORF2	138	NTF-2 family protein		-	-	-	-	-
ORF3	274	Hypothetical protein		-	-	66	-	-
ORF4	188	Adenylylsulfate kinase		-	-	-	-	-
SuoC	720	Oxygenase	AerC	-	59	-	-	-
SuoK	351	Type 2 isopentenyl-diphosphate Delta-isomerase	AerK	-	-	77	-	-
SuoD	202	Bacilysin biosynthesis protein BacA	AerD	-	63	63	63	75
SuoE	235	Hypothetical protein	AerE	-	53	47	53	60
SuoF	264	Short-chain dehydrogenase	AerF	-	71	68	68	70
SuoG	1637	Non-ribosomal peptide synthetase	AerG	-	60	56	60	72
ORF11	267	FabG, oxidoreductase		-	69	-	-	
SuoN1	595	ABC transporter	AerN	-	37	-	-	64
SuoH1	323	Dioxygenase	AerH	40	39	-	-	48
SuoH2	329	Dioxygenase	AerH	37	41	-	-	46
ORF15	374	Unknown			-	-	-	54
ORF16	499	MBOAT, acyl transferase		61	-	-	-	79
ORF17	612	Carbamoyl transferase			-	-	-	-
ORF18	135	SxtJ		-	-	-	-	-
ORF19	54	SxtK		-	-	-	-	-
ORF20	363	GDSL hydrolase		-	-	-	-	-
ORF21	322	Hypothetical protein		-	-	-	-	-
ORF22	303	Hypothetical protein		-	-	-	-	-
SuoH3	335	Dioxygenase	AerH	38	41	-	-	-
SuoH4	327	Dioxygenase	AerH	40	35	-	-	-
ORF25	277	Aldo-keto reductase		-	28	-	-	-
SuoI	419	Glycosyltransferase	AerI	62	57	-	-	-
ORF27	456	MFS transporter		47	-	-	-	-
SuoN2	673	ABC transporter	AerN	-	59	58	-	-

Supplementary Dataset 1 (separate excel file). The 103 aeruginosin biosynthetic gene clusters identified by bioinformatic analysis.
